# African agri-entrepreneurship in the face of the COVID-19 pandemic

**DOI:** 10.1186/s43170-023-00157-3

**Published:** 2023-06-01

**Authors:** Mariam A. T. J. Kadzamira, Adewale Ogunmodede, Solomon Duah, Dannie Romney, Victor Attuquaye Clottey, Frances Williams

**Affiliations:** 1 Centre for Agriculture and Bioscience International (CABI), Egham, UK; 2 CABI West Africa Centre, Accra, Ghana; 3 CABI Africa Centre, Nairobi, Kenya

**Keywords:** Firm size, Gender, Agri-SMEs, Agri-value chains, Africa

## Abstract

**Background:**

The African continent is known for high entrepreneurial activity, especially in the agricultural sector. Despite this, the continent's economic development is below expectations, due to numerous factors constraining the growth and sustainability of agricultural SMEs. These constraints have been exacerbated by the COVID-19 pandemic. The purpose of this study was to understand the pathways through which the pandemic affected agri-SMEs, with specific focus on assessing the differentiated effects arising from the size of the agri-SME and the gender of the owner-manager.

**Methods:**

Data was collected from over 100 agri-SMEs, ranging in size from sole proprietorships with one employee to agri-SMEs employing up to 100 people, in six African countries. Mixed methods were used to analyse the data with changes in business operations arising from changing market access, regimented health and safety guidelines and constrained labour supply assessed using visualisations and descriptive statistics. Logistic regression modelling was employed to determine the set of variables contributing to agri-SME business downturn during the COVID-19 pandemic.

**Results:**

All surveyed agri-SMEs were negatively affected by COVID-19-associated restrictions with the size of the firm and gender of the owner-managers resulting in differentiated impacts. The smallest agri-SMEs, mainly owner-managed by women, were more likely to experience disruptions in marketing their goods and maintaining their labour supply. Larger agri-SMEs made changes to their business operations to comply with government guidelines during the pandemic and made investments to manage their labour supply, thus sustaining their business operations. In addition, logistic regression modelling results show that financing prior to the pandemic, engaging in primary agricultural production, and being further from urban centres significantly influenced the likelihood of a firm incurring business losses.

**Conclusions:**

These findings necessitate engendered multi-faceted agri-SME support packages that are tailored for smaller-sized agri-SMEs. Any such support package should include support for agri-SMEs to develop sustainable marketing strategies and help them secure flexible financing that considers payment deferrals and debt moratorium during bona fide market shocks such as the COVID-19 pandemic.

## Introduction

The importance of the millions of small-and-medium-sized enterprises (SMEs) that source from smallholder farmers across Africa have been recognized by many as a catalyst for the growth of the agriculture sector (Reardon et al. 2019a, 2019b, 2019c). These SMEs, operating in the 'middle' of agricultural supply chains, provide and link smallholder producers to a wide variety of services, which include but are not limited to transportation, aggregation, logistics, processing and procurement. This has paved the way for support from governments and donors—who now see agri-SMEs as inclusive intermediaries that require support to reach scale and profitability in order to better serve smallholder farmers and contribute to development outcomes. Support has further been garnered from evidence showing that entrepreneurship can and does positively contribute to economic growth and development in Africa (Peprah and Adekoya 2020; Adusei 2016).

Such support for African agri-SMEs is essential because, despite the high entrepreneurial activity, the continent's economic development is below expectations (Ning 2021). This is due to numerous factors constraining African SMEs’ growth and sustainability. These include but are not limited to inadequate raw agricultural products, fluctuating international prices, poor infrastructure, poor managerial capital, bureaucratic government policies, inadequate financing (Nkwabi and Mboya, 2019; Igwe et al. 2018; van Klyton and Rutabayiro-Ngoga 2018; Asiedu et al. 2013). Finally, gender gaps in entrepreneurship (Pimpa 2021; Guzman and Kacperczyk 2019; Agyire-Tettey et al. 2018; Vossenberg 2013) as well as the interaction of gender roles with the developing country business environment (Ogundana et al. 2018), suggest that agri-SMEs owner-managed by women are further constrained, thus requiring more support. These constraints have been exacerbated by the COVID-19 pandemic.

Understanding the pathways through which African agri-SMEs have been impacted by the COVID-19 pandemic is essential for developing effective policy and investment responses. Currently, there is clear emerging evidence that the pandemic negatively affected food security, agriculture and livelihoods in the developing world (Workie et al. 2020). Specifically in Africa, emerging evidence shows that the pandemic worsened the challenges of the agriculture sector and negatively affected agricultural supply and value chains (Ali Mohamed et al. 2021; Hatab et al. 2021; Hirvonen et al. 2021; Hoyweghen et al. 2021; Nchanji et al. 2021; Ayanlade and Radeny 2020; Morton 2020; Sers and Mughal 2020). In addition, Apostolopoulos et al. (2021) examined 49 papers from the literature, which included some studies that involved or focused on Africa, that showed that globally, agri-food entrepreneurship had been negatively affected by the pandemic. Rigorous studies that provide insights on the differentiated effects of the COVID-19 pandemic as a result of the size of the enterprise and gender of the owner-manager of agri-SMEs are hard to find in the literature. This study contributes to filling this gap by providing gendered insights on how various sized agri-SMEs in several African countries across different value chains responded to changes arising in the market due to national COVID-19 restrictions. The findings of this study may contribute toward a better understanding of the differences in response by African agri-SMEs to market and supply chain disruptors such as the COVID-19 pandemic. Furthermore, the study can provide insights for governments concerned with creating a conducive business environment. At the same time it can aid the investor community in their efforts to develop innovative financing models for SMEs. Such efforts will ensure that African agri-SMEs are not 'hidden' from the policy arena or 'underserved' by the investment community, as elaborated by AGRA (2019) and CASA (2020), respectively.

## Study methodology

### Sample size and data collection

A telephone based survey of 119 market actors was conducted in mid-2020 (Zharare and Mashingaidze 2020). This included the collection of both qualitative and quantitative data from market actors who directly source from smallholder producers, in six countries across six different value chains (Table 1). The survey therefore interviewed aggregators, input suppliers, processors, export traders, seed producers and retailers. In addition, some of the sampled agri-SMEs also engage in primary agricultural production.

**Table 1:** Summary of market actor survey

Country	Value chain	Surveyed Market Actors	%age of sampled agri-SMEs by gender of owner-manager	
		Sample size	Man (%)	Woman (%)
Ethiopia	Rice	13	92%	8%
Ghana	Oil palm and Cocoa	29	59%	41%
Malawi	Groundnuts	9	78%	22%
Nigeria	Maize, Cocoa, Rice	24	79%	21%
Tanzania	Rice and Sunflower	26	73%	27%
Zimbabwe	Maize	18	83%	17%
**6 countries**	**6 value chains**	**119 surveys**		

Data collected from market actors included the gender of the agri-SME owner-manager, access to financing, number of employees (a proxy for the size of the enterprise), land size for those engaged in primary agricultural production, and distance from city/urban area and the age of business owner-manager. In addition, data was collected to understand changes in market access, financing, business operations and labour supply resulting from the COVID-19 pandemic and restrictions imposed to curb its spread.

### Data analysis

Data was analysed using mixed methods to determine how agri-SME business performance fared in the face of the COVID-19 pandemic and associated restrictions. Changes in business operations arising from changing market access, regimented health and safety guidelines and constrained labour supply were assessed using visualisations and descriptive statistics. To determine the set of variables contributing to agri-SME business downturn during the COVID-19 pandemic, the study employed logistic regression modelling. The logistic regression model has the advantage of producing more informative results than any other classification process because it describes the link between a predictor (or a set of predicator variables) and an outcome variable.

In this study, the outcome variable is a binary variable (whether or not an agri-SME suffers business losses), dependent upon a set of factors, which together influence the likelihood of surveyed agri-SMEs incurring business losses as a result of the COVID-19 pandemic. We postulate that these factors include the gender of the owner-manager, changes affecting the business due to the pandemic (market access, labour supply, financing), current levels of financing of the business, distance of the business operations from the nearest urban centre and land size for those engaged in primary agricultural production. We verified the data distribution using the “xy” scatter plot and estimated the value of kurtosis to determine whether to use the Logistic or Probit model (Sultana and Kiani 2011). Logistic regression was selected to investigate the factors impacting agri-SMEs business losses owing to the COVID-19 pandemic because the kurtosis value was positive. In its simplest form, this theoretical understanding of the factors causing agri-SMEs to suffer business losses during the pandemic can be represented as follows:

1 $${BL}_{i}={\beta }_{0}+{\sum }_{j=1}^{n}{\beta }_{i}{X}_{ij}+{\varepsilon }_{i}$$

where ${BL}_{i}$ is the dependent outcome variable with a value of 1 if the agri-SME suffered business losses as a result of the COVID-19 pandemic and 0 if otherwise. ${X}_{ij}$ is a vector of explanatory variables (see Table 2) and ${\varepsilon }_{i}$ is the error term.

**Table 2:** Model explanatory variables

Variable	Definition
Gender	Gender of owner-manager (1 = Female, 2 = Male)
Change in market access	Agri-SME experienced changes in market access (1 = Yes, 2 = No)
Current access to financing	Agri-SME currently has access to financing (1 = Yes, 2 = No)
Lockdown changes in accessing financing	Agri-SME experienced changes in financing due to the pandemic (1 = Yes, 2 = No)
Farm size	Farm size under cultivation for those agri-SMEs engaged in primary agricultural production (hectares)
Distance from city	Distance of agri-SME operations from urban centre/city (Kilometres)
Change in labour supply	Agri-SME experienced changes in labour supply (1 = Yes, 2 = No)

## Results

### Profile of surveyed agri-SMEs

**Fig. 1:**
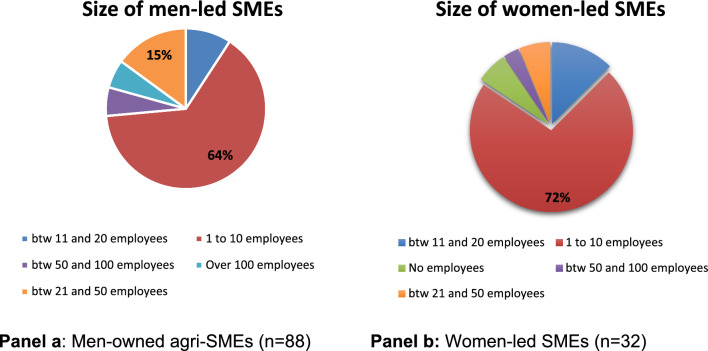
Size of sampled agri-SMEs.

Twenty-seven percent of the 119 agri-SMEs surveyed were women-owned enterprises. However, differences were observed between countries (see Table 1), with Ghana having the highest proportion of women-owned agri-SMEs (41.3%) and Ethiopia the lowest (7.69%) (Fig. 1). These statistics agree with other findings, which show that the ratio of male to female entrepreneurship differs across different countries in Africa, with men more likely to engage in entrepreneurship than women (UNECA 2019; Herrington and Kelly, 2013). These disparities can also be attributed to the sampling of different value chains, with women's engagement in agricultural entrepreneurship differing between different value chains across different countries (Brenton et al. 2013). Differences aside, statistics from across the continent show that sub-Saharan Africa has the highest female entrepreneurship rate globally (Campos and Gassier 2017, Herrington and Kelly, 2013). However, these women-led enterprises are more likely to be smaller, informal, and less profitable, with less growth potential than those operated by men (Agyire-Tettey et al. 2018; Campos and Gassier 2017; Terjesen and Lloyd, 2015).

Table 3 provides a summary of the profile of sampled agri-SMEs. From the summary, it is clear that there is gender parity in terms of the age of the owner-manager amongst sampled agri-SMEs. This agrees with global statistics, which show that in low-income countries, there is gender parity in age for entrepreneurs (GEM, 2022). Table 3 also shows that, on average, most surveyed agri-SMEs operate on the outskirts of an urban area (i.e., within 50 km from the city). The average land holding for those in the sample that are involved in primary agricultural production (approximately 20% of the sample) is 40 hectares and 31 hectares for men and women-owned SMEs, respectively. This makes them part of the growing body of the commercially oriented African medium-scale farmers, who cultivate or own, on average, between 5 and 100 hectares of land (Jayne et al. 2016). Twenty-one percent of surveyed agri-SMEs had access to finance prior to the pandemic. Finance includes loans from commercial banks, loan sharks, microfinance institutions, private investment funds and remittances from individuals in the diaspora as well as government and private contract farming schemes.

**Table 3:** Profile of surveyed market actors

	Male-owner (n = 88)	Female-owner (n = 32)	
Average age o agri-SMEs owner-manager (years)	43	42.9	
Average distance from nearest city/urban center (km)	50	46	
Percentage of market actors owning/leasing farm land(%)	24%	31%	
Average farm land for those cultivating land (ha)	40	31	

Almost all surveyed agri-SMEs (93.2%) employ other people in their business, with an average of 20 employees per business. Larger agri-SMEs, in terms of the number of employees, tend to be owner-managed by men (Fig. 1). This is the case with the majority of agri-SMEs owner-managed by women falling within the smallest sized category of SMEs (i.e., those with between 1 to 10 employees).

### Changes in business operations due to the pandemic

All countries where data was collected for this study implemented different restrictions to curb the COVID-19 pandemic; see Haider et al. (2020) and Umviligihozo et al. (2020) for an overview of lockdown measures implemented in Africa. From the market survey, we observed that the majority of agri-SMEs, regardless of the size of the enterprise, made changes to their business operations as a response to different national COVID-19 restrictions. This implies that the effects of the COVID-19 pandemic affected all agri-SMEs, regardless of the size of operation. This finding agrees with other studies which show that African SMEs in general, regardless of size, were affected in some ways by the pandemic (Ojong-Ejoh et al. 2021; Amoussou et al. 2021).

**Fig. 2:**
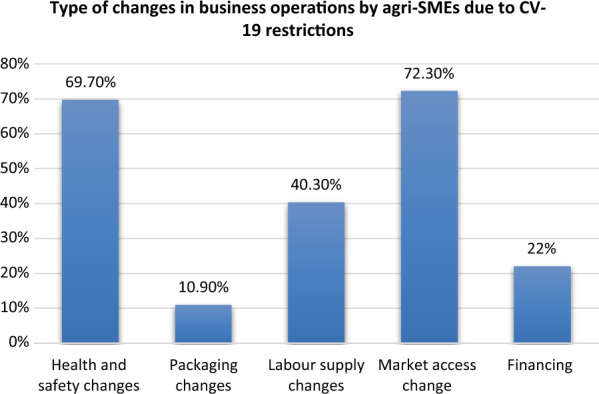
Changes in business operations experienced by all surveyed agri-SMEs

Specifically most surveyed agri-SMEs made changes that affected the marketing of their goods (72.3%). In addition, just under 70% of all surveyed agri-SMEs also made changes related to health and safety standards of their business premises. Amongst the sample, a few agri-SMEs stated that they experienced labour supply shortages (40%) and some had disruptions in their financing (22%) (Fig. 2).

Changes in market access varied but were all attributed to lockdown measures that reduced or banned travel and movement of goods and people between different localities. Larger agri-SMEs responded to this by hiring transport to ferry their produce to available markets and/or end customers. In terms of gender, agri-SMEs owner-managed by women were more likely to experience changes in market access as compared to those owner-managed by men. This was the case with 80% of all sampled women owner-managed agri-SMEs compared to 72% of SMEs owner-managed by men experiencing changes in market access due to the pandemic.

Amongst the survey respondents, the larger agri-SMEs were less likely to have trouble with market access during the pandemic. For example, our data shows that 83% and 85% of agri-SMEs with between 21 to 50 and those with over 100 employees stated that they not did experience any change in marketing of their goods. This can be attributed to larger agri-SMEs having structured markets and contracts – which were not altered due to the pandemic. This is in contrast to the smaller agri-SMEs, with the majority (70%) of those with less than 20 employees having had difficulties with accessing markets for their goods during the pandemic. Smaller agri-SMEs survived during the pandemic by tapping into their own local networks and selling only to customers within their residential areas. This agrees with a study from South Africa, which showed that networks were key for SME survival during the pandemic (Fubah and Moos 2022).

Many of the larger agri-SMEs made changes to their health and safety standards to comply with government directives, thus allowing them to continue business operations, and to fulfil their contracts. This included all surveyed agri-SMEs having 50 to 100 employees and most (67%) of those with over 100 employees. Agri-SMEs owner-managed by men (67%) and women (74%) were as equally likely to make changes in the health and safety standards of their business operations. Changes included innovative packaging, the wearing of face masks, installing hand washing stations and practicing social distancing. Smaller SMEs were less likely to put in place these measures. This can be attributed to the additional costs of business operations that emanated from enacting these measures. A few of the surveyed agri-SMEs put in place additional measures, which included the installation of foot baths, use of gloves by staff handling produce, periodic fumigation of business premises and temperature checking of customers upon entry into the business premises. In addition, some enterprises shifted towards electronic payments and customer/visitor logs. For those sourcing from farmers, agricultural produce weighing and verification were done through a window rather than the farmer or cooperative representative entering the office. Anecdotal evidence of farmer dissatisfaction with these market changes and resultant lack of transparency have been documented, but no rigorous evidence exists. Costs associated with implementing various health and safety-related standards were paid for either by staff contributions or were transferred to the final consumer.

About 40% of all agri-SMEs surveyed experienced changes in labour supply—either on the supply side and/or on the demand side. Of these, more of the agri-SMEs owner-managed by women (45%) were more likely to experience labour supply constraints as compared to those that are owner-managed by men (35%). The constraints varied, on the supply side, there were fewer labourers available, and those that were available demanded higher wage rates. These findings are similar to findings from Nchanji et al. (2021), Ratnasingam et al. (2020) and Shafi et al. (2020). On the demand side, SMEs reduced operations, thus requiring fewer workers. In addition, surveyed agri-SMEs asked casual workers to stop attending sites, while larger enterprises shifted to varying work schedules. In rare cases, larger surveyed agri-SMEs, hired nurses and additional security personnel to support them with enforcing restrictions on social distancing and mask-wearing. This was observed only for those agri-SMEs with more than 100 employees and having large factory facilities. So in general larger enterprises were less likely to perceive labour supply as an issue during the pandemic. This is because most of the large enterprises made adjustments that included providing transport for their staff to attend shifts in the absence of public transport. Smaller enterprises, on the other hand, perceived labour shortages as being a major problem during the pandemic. This can be attributed to that larger agri-SMEs were more likely to have cash or financial resources, which enabled them to make adjustments to sustain their labour supply. This concurs with findings from the corporate sector, which demonstrates that corporate firms with cash or larger cash reserves outperformed their counterparts without cash or with fewer cash reserves during the COVID-19 pandemic (Zheng 2022).

All the changes made and experienced by agri-SMEs as stipulated above, as well as limited business operations due to the various COVID-19 restrictions led to increased operational and transactional costs for surveyed agri-SMEs. This resulted in the majority (89%) of the smaller surveyed agri-SMEs self-reporting business losses. This concurs with other studies which showed that during the pandemic, agri-SME/SMEs experienced higher operating costs (Lakuma et al. 2020; Muriithi 2021). However, a few of the surveyed agri-SMEs in the smaller size category (11%) self-reported higher profits during the pandemic. This they attributed to higher market prices of their goods, panic buying by consumers and fewer enterprises operating in the market. In the absence of proper financial records, it was not possible to quantify the change in profits or to determine if the self-reported profits were sustained beyond the short term.

### Logistic regression model results

Table 4 presents results of the logistic regression model to assess whether surveyed agri-SMEs suffered business losses during the pandemic. Access to financing, engaging in primary agricultural production and being further from urban centres significantly influence sampled agri-SMEs incurring business losses. Access to financing exhibits a positive association with business losses. This means that agri-SMEs that had a business loan pre-pandemic, i.e., agri-SMEs with repayment obligations, were more likely to incur losses during the pandemic. In our study, this finding is attributed to agri-SMEs being obligated to repay loans even in the face of reduced business operations. Ordinarily, financing helps firms to innovate and to overcome challenges. However the severe impacts of restrictions associated with the COVID-19 pandemic, resulted in SMEs losing liquidity in a short period of time, in which they were unable to renegotiate loan repayment terms. This finding agrees with emerging evidence from the IMF (2021). Some studies however contradict our findings as they show that small firms, in Africa or other parts of the world, that are constrained by access to credit, were more vulnerable to the effects of the COVID-19 pandemic (Khan 2022; Zhang and Sogn-Grundvag 2022).

**Table 4:** Has business suffered losses as a result of the COVID-19 pandemic (1-Y, 2-N)

Variable	Business loss
	Coefficients
Gender	− 0.430 (0.488)
Change in market access	0.619 (0.506)
Current access to financing	1.155** (0.496)
Lockdown changes in accessing financing	0.564 (0.488)
Farm size (ha)	0.0115* (0.00646)
Distance from city (km)	− 0.0145*** (0.00437)
Change in labour supply	0.259 (0.486)
Constant	− 1.419** (0.641)
*LR chi*^*2*^*(7)*	*30.12*
*Prob* > *chi*^*2*^	*0.0001*
*Pseudo R*^*2*^	*119*

*** p < 0.01, ** p < 0.05, * p < 0.1. Standard errors in parentheses. Model LR chi^2^ (7) = (30.12); Prob > chi^2^ = 0.0001

Farm size exhibits a positive association with business losses. This implies that agri-SMEs that had cultivated larger pieces of land were more likely to incur losses during the pandemic than those that had cultivated smaller pieces of land. This can be attributed to labour supply shortages, resulting in higher wage rates or the lack of labourers resulting in crops being damaged in the field during harvest time. Such incidences have been documented by, for example, (Zharare and Mashingaidze 2020). However, in this study, it is likely that losses were incurred because most of the surveyed agri-SMEs, that engaged in primary agricultural production, were also linked to the agri-food manufacturing industry. Emerging evidence has shown that the agri-food manufacturing sector in Africa was significantly and negatively affected by the pandemic and associated restrictions (Oman et al. 2021). Therefore feeder industries, such as surveyed agri-SMEs in this study, would also be significantly and negatively affected.

Distance from an urban center exhibits a negative association with business losses. This implies that agri-SMEs that were further away from an urban center or city were more likely to incur losses than agri-SMEs located closer to urban centres. This might be expected given that most lucrative markets are located closer to cities. Numerous anecdotal evidence concurs with our findings and shows that the COVID-19 pandemic had differential effects on rural and urban economies, with rural economies more negatively affected. However, findings by Maredia et al. (2022), contradict our findings, showing that the impacts of the COVID-19 pandemic on African rural and urban incomes were essentially similar.

## Conclusion and recommendations

Evidence from six African countries from various market actors operating in six different value chains, clearly shows that smaller-sized agri-SMEs, mainly owned by women, were more likely to experience disruptions in the marketing of their goods and more likely to experience problems with labour supply in the face of restrictions to curb the COVID-19 pandemic. Consequently, these smaller agri-enterprises were also less likely to put in place health and safety mechanisms to protect their staff and customers from further spread of the COVID-19 virus or to invest resources to secure their labour supply. These factors combined led to the majority of the smaller agri-SMEs, experiencing financial losses as a result of the pandemic. Larger agri-SMEs, on the other hand, were more likely to shift funds to adapt their operations in response to the pandemic. This included adaptations to business operations to safeguard the health and well-being of staff and customers and investments to ensure and sustain their labour supply. Agri-SMEs operating further from urban centres with loan obligations and those engaged in primary agricultural production were also more likely to incur business losses.

A key recommendation is that public and private sector entities facilitating entrepreneurship development should establish multi-faceted support packages that will cushion agri-SMEs in the aftermath of the COVID-19 pandemic to allow them to bounce back. Support should be tailored for smaller-sized agri-SMEs as they are more vulnerable to shocks arising in the market. In addition, there is a need for innovative gendered support packages, as most enterprises owner-managed by women are likely to be smaller and hence more vulnerable. Any support package put in place should have a work package to support agri-SMEs with developing sustainable marketing strategies and securing flexible financing that considers payment deferrals and debt moratorium for agri-SMEs in the event of a bona fide economic and/or market shock. Smaller-sized agri-SMEs should be supported not only with financing but also with the capacity to diversity their business operations and income streams to enhance their resilience in the event of market and economic shocks such as the COVID-19 pandemic.

Future areas of research should focus on exploring how agri-SMEs self-reporting profits during the pandemic exploited digitization, innovation and new market opportunities as compared to their counterparts who self-reported losses. In addition, given disparities in the literature, there is need to continue exploring the impacts of the pandemic on agri-SMEs based in different localities i.e. rural vs urban areas.  Finally there is need for research to quantify the specific impacts of the COVID-19 on different agri-SMEs and their recovery rates, given their backward-and-forward linkages with various agri-food manufacturing industries.   

## Data Availability

Not applicable.
